# Health Evaluation in the Context of Satisfaction with Medical Services among Patients with Osteoarthritis: Descriptive Cross-Section Survey

**DOI:** 10.3390/ijerph17010009

**Published:** 2019-12-18

**Authors:** Ewelina Firlej, Mariola Janiszewska, Katarzyna Sidor, Anna Sokołowska, Agnieszka Barańska, Paweł Chruściel

**Affiliations:** 1 Cosmetology and Aesthetic Medicine Unit, Faculty of Pharmacy with Medical Analytics Division, Medical University of Lublin, 20-093 Lublin, Poland; ewelinafirlej@umlub.pl (E.F.); anna.sokolowska@umlub.pl (A.S.); 2 Department of Medical Informatics and Statistics with E-learning Lab, Faculty of Health Sciences, Medical University of Lublin, 20-081 Lublin, Poland; mariolajaniszewska@umlub.pl (M.J.); agnieszkabaranska@umlub.pl (A.B.); 3 Departament of Applied Psychology, Chair of Psychology, Interfaculty Centre for Didactics, Medical University of Lublin, 20-093 Lublin, Poland; katarzyna.sidor@umlub.pl; 4 Department of Basic Nursing and Medical Teaching, Chair of Development in Nursing, Faculty of Health Sciences, Medical University of Lublin, 20-081 Lublin, Poland

**Keywords:** osteoarthritis, medical services, rehabilitation clinic, seniors

## Abstract

Osteoarthritis (OA) is one of the most common causes of rehabilitation benefits and senior disability. It generates high costs of treatment and increasing demand for medical staff and care of geriatric profile. The aim of the study was to determine the relationship between health evaluation and satisfaction with medical services among individuals with OA in rehabilitation outpatient clinics. The survey was carried out from June 2017 to May 2018, among patients being provided with services of five outpatient rehabilitation clinics in Lublin. The surveyed group comprised 328 respondents. The following tools were utilized: the List of Health Criteria (LHC), the Multidimensional Health Locus of Control Scale (version B) (MHLC), the authors’ own questionnaire compiled for the study, and the Servperf Method. According to the respondents, the most important health criterion is “not to experience any ailments” (M = 1.56). In an assessment of a clinic, the respondents rated neatness (cleanliness) of the staff highest (M = 4.38) and the appearance of a building where a clinic is located lowest (M = 3.42). The better the evaluation of medical services in an outpatient rehabilitation clinic in comparison to other settings, the better the evaluation of the quality of service (rho S = 0.593; *p* < 0.000). The study conducted in outpatient rehabilitation clinics showed great demand for outpatient specialist care of geriatric profile. Undoubtedly, there is need for continuation and expansion of studies on patients with OA in other rehabilitation settings.

## 1. Introduction

Osteoarthritis (OA) (Latin: arthrosis, osteoarthritis, osteoarthrosis) constitutes a common cause of necessity for using rehabilitation benefits by seniors [1,2,3]. It is the most common disease of the locomotor system, and its prevalence increases with age [4,5,6]. The definition of OA has not been determined yet. Experts of the American Academy of Orthopaedic Surgeons, the National Institute of Arthritis, Musculoskeletal and Skin Diseases, the National Institute on Aging, the Arthritis Foundation, and the Orthopaedic Research and Education Foundation compiled the following definition of OA: Osteoarthrosis is determined by the effect of mechanical and biological factors that influence the process of degradation and synthesis of joint cartilages and subchondral bone layer. It concerns all tissues of the joint. OA is a cause of softening, fibromatosis, ulceration and depletion of articular cartilage. It also causes hardening and thickening of the subchondral bone, osteophytes creation and subchondral bone cysts. Clinical symptoms of the disease include arthralgia, pain on pressure, restricted motion, crackling noise, effusion, and inflammation [7]. The onset is slow. With time, some depressions and fissures become visible on the surface of the cartilage. Advanced (OA) is characterized by complete destruction of cartilage and exposure of the articular surface [8,9,10]. The World Health Organization (WHO) states that OA symptoms occur in 9.6% of the world male population and 18% of the world female population at the age of 60. With reference to Europe, this constitutes 10–15% of the sick.

According to the WHO, OA belongs to the group of civilization diseases that threaten health of the population [11,12]. Progression of the disease results in an increase in treatment costs and growing need for medical staff and specialized care, particularly of the geriatric profile [13,14]. According to the Organization for Economic Co-Operation and Development (OECD), costs that were borne by hospital and outpatient care constitute a substantial part of the expenditure among the members of the OECD (nearly two-thirds of the expenses for health). Unfortunately, the costs considerably exceed other expenses for health, including outpatient medical benefits, medicines, and long-term care. It has a direct relationship with ways and quality of medical needs fulfilment in patients with OA. Expenses for outpatient care, including rehabilitation, are too low in relation to the number of patients with OA. This leads to increasing queues of patients waiting for appointments in rehabilitation clinics and lack of appropriate equipment, as well as adjustment of room infrastructure in settings providing outpatient services. Patients of most membership countries of the OECD positively evaluated the amount of time doctors devoted to them during appointments, including explaining medical terms and their engagement in the whole process of treatment. At the end of 2017, the population of Poland amounted to 38.4 million, out of which over nine million were persons aged over 60 (>24%). The population of the Lubelskie Province amounted to 2,133,300, including 510,200 persons aged over 60. Within the Lubelskie Province area, the city of Lublin has one of the highest shares of the elderly in the total number of inhabitants [15,16]. The report conducted in Poland showed the highest rates of the abovementioned parameters. Poles are of the opinion that doctors do not devote enough time during an outpatient appointment and do not involve themselves appropriately in the process of care and treatment (along with clarifying medical terms) [15,16,17,18]. In the face of current demographic prognoses and consistent increase in the number of seniors with functional restrictions and disability, there is greater requirement for complex rehabilitation services. Roughly 80 million of people are excluded from active social and professional life. OA creates a considerable decrease in the quality level of a patient’s life and their daily fitness. The Eurostat data of 2016 show a proportional dependence between age and chronic restrictions (73.3% in a group of 85-and-more-year-old people). The findings of the European Health Interview Survey demonstrated that sensory and physical functional barriers were mentioned by over two-thirds of the respondents in a group of 65-and-more-year-old people. Among this community, more than one-fifth of them have difficulties with self-care and daily performance [19,20,21,22].

Changes in medical-care systems that concern reducing the costs of outpatient health benefits in favor of hospital treatment influence quality and availability of medical care among people with OA. The way patients with OA perceive health, whom they attribute control over their health to, and how they evaluate medical services are questions of great significance in the context of improvement of health situation in the group of patients. Health evaluation in the context of satisfaction with medical services contributes to the innovative dimension of this paper. Satisfaction is the outcome of the quality of the available services, which either meet patients’ expectations or are deficient in this respect. Satisfaction with medical services is influenced by the relationship between the patients and medical staff. The paper discusses patients’ evaluation of the medical staff’s knowledge and conscientiousness and politeness toward patients: the opening hours of outpatient clinics, the availability of medical equipment, the willingness of the medical staff to help, the proximity of outpatient clinics, the accessibility of outpatient clinics for persons with disabilities, cleanliness in outpatient clinics, neatness (cleanliness) of the medical staff, the readability of outpatient clinics’ websites, the appearance of outpatient clinic buildings, emergency services, and the understanding and patience of the medical staff toward patients. The assessment of the correlation between the values the patients attribute to health and their evaluation of medical services provided in Lublin-based outpatient clinics represents an important research contribution. This correlation is examined here also in relation to the increasing number of seniors suffering from degenerative changes and experiencing serious limitations in access to rehabilitation services in Lublin. We tested a hypothesis assuming that there was a correlation between the evaluation of health and satisfaction with medical services in seniors suffering from osteoarthritis.

## 2. Materials and Methods

### 2.1. The Sample Selection

The study was carried out among patients being provided with rehabilitation services in outpatient rehabilitation clinics in Lublin, Poland. Those settings were as follows:

▪ The Rehabilitation Clinic at St. John of Dukla Oncology Center of the Lublin Region;
▪ The Rehabilitation Clinic at the Institute of Rural Health;
▪ The Independent Public Healthcare Setting of the Ministry of the Interior and Administration;
▪ The Neuropsychiatric Hospital of Professor Mieczysław Kaczyński;
▪ The Independent Public Clinical Hospital No.4.

The population of Lublin was surveyed, since this city is characterized by a substantial number of patients with OA in Eastern Poland. Moreover, according to the Central Statistical Office, one of the highest rates of elderly population is found in Lublin.

The number of 60-year-old-and-more patients with OA diagnosed who were provided with outpatient rehabilitation care amounted to 362 individuals in Lublin. The size of the sample was 347 respondents (*d* = 5%; CI = 95%). Because of lack of some data in the questionnaires, 19 were rejected. Finally, 328 respondents took part in the survey: 266 females and 62 males. The selection of the study group was based on both world data showing that OA is the most common disease of joints, particularly in individuals over 60 years of age, and epidemiological data regarding frequency of appointments in rehabilitation centers in Poland. The study was approved by the Committee of the Bioethics at the Medical University of Lublin (no. KE-0254/124/2017).

Nonprobability sampling was applied based on availability of respondents at a particular time. Prior to filling in the questionnaires, each patient was informed about the purpose of the study and conditions of its course, such as lack of time limit for filling out questionnaires, lack of participation fees in the study, full anonymity, and freedom of participation. Each patient was asked to read and sign the consent form before filling in and signing information regarding the survey form. The range of materials collected was satisfactory, though there were some difficulties connected with completing the questionnaires because of the patients’ age and specific course of the disease. The questionnaires were completed individually, in the presence of a pollster.

### 2.2. Respondent Selection Criteria

The study included patients who gave their informed consent to take part in the study, were at least 60 years old, were treated for osteoarthritis, and used rehabilitation services in rehabilitation outpatient clinics. Individuals who did to meet the above criteria were excluded from the study.

### 2.3. Instruments

The diagnostic survey along, with the following questionnaires, were applied in the survey:

(1) The List of Health Criteria (LHC) by Z. Juczyński that consists of 24 items determining health in the physical, mental, and social dimensions. The reliability coefficient of the LHC was measured, using the test-retest method. It was determined at 0.68, which may be considered satisfactory. The questionnaire enables reference to the meaning of the word “health” and what a respondent means by “being healthy”. Getting to know respondents’ opinions within the abovementioned is of great significance in pro-health activities. Diversity of the definition of health and ways of health measurement hinder or prevent activities directed at health protection, enforcement, and restoration. The tool is used to assess adults, both the healthy and the sick; a study can be conducted individually or in a group. The LHC refers to holistic comprehension of health. In the study, respondents chose the most important criteria of health in the column A, and then they selected only five most significant ones in the column B, out of those marked in A. The last stage concerns prioritization of health criteria in the column C, attributing them values from 5—the most important to 1—the least important. In analyzing the results, special attention was paid to definitive properties of health understood as status, process, property, and outcome [23].

(2) The Multidimensional Health Locus of Control Scale (MHLC) version B, Polish adaptation by Z. Juczyński, is composed of 18 items that assess respondents’ subjective beliefs concerning health. Reliability was determined on the basis of Cronbach’s alpha. In regard to version A, it was 0.74 for internal locus of control, 0.69 for chance locus of control, and 0.54 for “external” locus of control. The structure of the tool is based on an assumption that internal health locus juxtaposes with external one by differentiating the latter as dependent on an influence of others or an influence of coincidence. The questionnaire distinguishes three dimensions of health control:

a. Internal Health Locus of Control (IHLC)—controlling someone’s health depends on this particular person.

b. External Health Locus of Control (PHLC)—someone’s health results from an influence of people, especially medical staff.

c. Chance Health Locus of Control (CHLC)—chance or other external factors determine someone’s health status.

A respondent attributed each item with a value on a 6-point scale: from point 1—I definitely disagree, to point 6—I definitely agree. Results of each of three dimensions were calculated separately by adding points. The results range from 6 to 36 points, which means the higher the result, the stronger the conviction of an influence of a particular factor on health [23].

(1) Service Performance (Servperf, SP), Polish adaptation by R. Wolniak, is a different method of SERVQUAL that is used to assess the quality of medical services provided by an outpatient clinic. In regard to the variables defined in the study, Cronbach’s alpha values exceeded 0.7 in each instance. The reliability, which was assessed by using the test–retest method (after six weeks), proved better for Version B (IHLC scale *r* = 0.72, PHLC scale *r* = 0.64 and CHLC scale *r* = 0.60). The questionnaire includes a set of 14 variables, as well as four factors of quality of services:

a. The quality of service (8 variables are found for a factor, and they are as follows: V1—conscientiousness of the medical staff toward patients; V2—knowledge of the medical staff, expertise of the medical staff; V3—politeness of the medical staff during a service; V4—opening hours of an outpatient clinic; V5—medical equipment available; V6—willingness of the medical staff to help, V13—services provided in emergency; V14—understanding and patience of the medical staff toward patients).

b. Physical aspects (4 variables are included: V8—adopting an outpatient clinic for the disabled, V9—cleanliness in an outpatient clinic, V10—neatness (cleanliness) of the medical staff, and V12—the appearance of a building where an outpatient clinic is located).

c. E-outpatient clinic (only one variable: V11—assessment of an outpatient clinic’s website)

d. Localization (one variable is found: V7—the proximity of an outpatient clinic to patients’ place of residence).

Variables regarding the perception of quality of services were assessed with a 5-point Likert scale: 1—very bad, 2—bad, 3—medium, 4—good, and 5—very good. For each variable and hidden factors of quality of services, mean values were calculated, and they ranged from 1 to 5 [24].

(1) The authors’ questionnaire compiled for this particular study comprises 17 questions referring to the following:

a. Evaluation of health;

b. Risk factors;

c. Diagnostics;

d. Prevention;

e. Methods of treating OA.

Sociodemographic data (age, gender, civil status, place of residence, education, living conditions, and source of maintenance).

### 2.4. Data Analysis

The analysis of results obtained was performed by using IBM SPSS Statistics 19 (IBM, Kraków, Poland) and Statistica 10 (StatSoft, Kraków, Poland). Spearman’s rho was applied to check if there were statistically significant correlations between quotient variables and ordinal variables. Prior to the tests, a normality test was conducted, using the Shapiro–Wilk test. The level of statistical significance was *p* < 0.05. Since almost all the variables subject to statistical analysis were ordinal or quasi-quotient, and the compared groups were characterized by significant disproportions in terms of quantities, the normality of distributions of the analyzed variables was not examined, and we assumed that nonparametric tests and coefficients would be applied.

## 3. Results

Having analyzed the demographic data obtained, the mean age of the patients was 67.90 ± 6.70. Among the respondents, as many as two out of three (66.2%) were married. A total of 28.4% constituted widows and widowers. Taking into account the place of residence, most of the respondents lived in the urban areas. Almost half of the individuals surveyed (46.6%) had secondary school education, 29% higher education, 19.8% vocational one, and solely 4.6% primary one. A half of the respondents (50.9%) assessed their living standards as good; every fourth individual (25.9%) assessed them as average and 23.2% as very good. Among the patients, three out of four (75.6%) lived on a pension or retirement pension. More than half of the respondents (58.2%) assessed their health status as average and 23.2% as very good. Every fifth person (20.7%) was of an opinion that his/her health was good; fewer respondents assessed it as bad (12.8%), for 5.2% it was very bad, and for 3% it was very good. As many as 70.1% of the respondents admitted that health is the greatest value for them. A total of 22% of them partially agreed with the statement, 7.3% was of a different opinion, and 0.6% had no opinion.

The detailed health criteria outlined in the LHC are part of individual general health dimensions: state, purpose, process, property, and outcome. The analysis of responses to individual detailed questions in the LHC showed that the most important health criterion as perceived by the respondents was “not to experience any ailments” (M = 1.56) This was followed by “to live up to old age” (M = 1.49), “to have all parts of the body functional” (M = 1.39), “to follow a healthy diet” (M = 1.25), “almost never to have to see a doctor” (M = 1.12), “to get on well with other people” (M = 0.91), and “to feel happy most of the time” (M = 0.79).

It was found that in terms of the general dimensions of the LHC, health was defined by the study participants largely as a property (M = 0.76): “to have all the body parts functional” (property), “almost never have to see a doctor” (property), “not to get sick, except for occasional flu” (property), “not to experience any ailments” (property), “to have healthy eyes, hair and complexion” (property), “to be in a good mood” and “to take medications only exceptionally” (property). Similar results were obtained for the respondents’ perception of health as a state (M = 0.67): “to feel happy most of the time” (state), “to be able to enjoy life “ (state), “to feel good” (state), as a purpose (M = 0.65): “to accept oneself, to know one’s own abilities and deficiencies” (purpose), “to be responsible” (purpose), “to live up to old age” (purpose), and as an outcome (M = 0.58): “to follow a healthy diet” (outcome), “not to smoke cigarettes” (outcome), “to make sure to get enough rest and sleep” (outcome), “to have a job, varied interests” (outcome), “to have an appropriate body weight” (outcome), and “to drink little or no alcohol” (outcome). The respondents were the least likely to perceive health as a process (M = 0.45) (to be able to control one’s own emotions and drives, to get on well with people, to be able to work without tension and stress, to be able to adapt to changes in life, to be able to solve one’s own problems).

In the survey of satisfaction of the elderly suffering from OA with the services provided by an outpatient rehabilitation clinic, neatness (cleanliness) of the medical staff was assessed highest, and the appearance of a building where an outpatient clinic is located was assessed lowest. Since all the aspects were provided with mean values above 3.0, they are noteworthy. Neatness (cleanliness) of the medical staff was provided with an assessment of between good and very good. Cleanliness in an outpatient clinic, opening hours of an outpatient clinic, politeness of the medical staff, willingness of the medical staff to help, expertise of the medical staff, knowledge of the medical staff, conscientiousness of the medical staff toward patients, understanding and patience of the medical staff toward patients, assessment against other outpatient clinics, readability of an outpatient clinic’s website, and medical equipment available were well-assessed. Assessments, between good and medium were attributed with the remaining aspects, namely adopting an outpatient clinic to the disabled, the proximity of an outpatient clinic to patients’ place of residence, providing services by an outpatient clinic in emergency, and the appearance of a building where an outpatient clinic is located. The individual aspects of the assessment of outpatient clinics comprise four general dimensions. Taking into account general dimensions, the total quality of service (M = 3.99), physical aspects (M = 3.93), and E-outpatient clinic (M = 3.89) were assessed, well. Localization of an outpatient clinic (M = 3.65) was given between good and medium assessment (Table 1 and Table 2). The study proved that the higher an assessment of an outpatient clinic against other settings, the higher its assessment in terms of the quality of service provided in it (rho S = 0.593; *p* < 0.000), physical aspects (rho S = 0.411; *p* < 0.000), E-outpatient clinic (rho S = 0.341; *p* < 0.000), and localization (rho S = 0.155; *p* < 0.005). In their assessments of outpatient clinics, the respondents drew on their experiences of care received in other clinics, which determined all the assessment aspects. What is interesting is the fact that frequency of being provided with services is correlated only with the total quality of service (rho S = 0.214; *p* < 0.000). The more frequent services in an outpatient clinic are provided, the higher the assessment of the quality of service there.

With reference to general dimensions of health, stronger IHLC (I am in control of my own health), was proved to enhance the significance of health understood as a purpose (rho S = 0.194; *p* < 0.000) and outcome (rho S = 0.272; *p* < 0.000). The majority of health control as an influence of others (PHLC) (in particular, the influence of the medical staff on one’s state of health) raises the rank of health understood as a purpose (rho S = 0.178; *p* < 0.001) and diminishes the role of health as a property (rho S = −0.202; *p* < 0.000) (Table 3).

The stronger the sense of having control over one’s health (internal locus of control), the more important are such criteria as to live up to old age (rho S = 0.212; *p* < 0.000), to make sure to get enough rest and sleep (rho S = 0.261; *p* < 0.000) and not to smoke cigarettes (rho S = 0.192; *p* < 0.000). However, the stronger the IHLC, the less important such criteria as not to get sick, except for occasional flu or indigestion (rho S = −0.198; *p* < 0.000) and to have all parts of the body healthy (rho S = −0.225; *p* < 0.000). Stronger PHLC (one’s health is influenced by the actions of others, in particular the medical staff) causes a decrease in significance of health criteria: almost never have to go to see a doctor (rho S = −0.220; *p* < 0.000) and enhances the perception of health as to live up to old age (rho S = 0.231; *p* < 0.000). The stronger CHLC (the state of health is influenced by chance events or other external factors), the more frequent the perception of health as being in a good mood (rho S = 0.170; *p* < 0.002), and drinking little or no alcohol (rho S = 0.151; *p* < 0.006) as well as a decrease in significance of the criterion to have all parts of the body healthy (rho S = −0.145; *p* < 0.008) (Table 4).

The detailed analysis of the correlations between the individual LHC criteria and satisfaction with services (four general dimensions of the SP) showed that the higher the assessment of the overall quality of services (conscientiousness of the medical staff toward patients, the expertise of the medical staff, the politeness of the medical staff, the opening hours, the medical equipment available, the willingness of the medical staff to help, emergency medical services, the understanding and patience of the medical staff toward patients), the greater importance of the health criterion “to accept oneself, to know one’s own abilities and deficiencies” (rho S = 0.224; *p* < 0.000). However, the higher the assessment of the quality of service, the less important the perception of health as drinking little or none alcohol (rho S = −0.257; *p* < 0.000). In addition, the higher the assessment of the outpatient clinic in physical terms (the accessibility of the outpatient clinic for persons with disabilities, cleanliness in the outpatient clinic, neatness (cleanliness) of the medical staff, the appearance of the clinic’s building), the more important the health criterion “to be able to enjoy life” (rho S = 0.211; *p* < 0.000). Higher assessment of an outpatient clinic in terms of material aspects exposed a decrease in significance of health criteria of being able to solve one’s own problems (rho S = −0.193; *p* < 0.000) and not experiencing any ailments (rho S = −0.229; *p* < 0.000). Higher assessment of an E-outpatient clinic (the assessment of the outpatient clinic’s website) determines the perception of health as being able to enjoy life (rho S = 0.332; *p* < 0.000). However, better assessment of localization of an outpatient clinic (the proximity of the outpatient clinic to the patient’s place of residence) shows greater significance of health perceived as being able to work without tension and stress (rho S = 0.231; *p* < 0.000) (Table 5).

## 4. Discussion

The terms, “health” and “disease” are very comprehensive. As the literature shows, they are interpreted in many different ways. Currently, health is considered to have a multidimensional character. This is particularly evident in a definition of health by the WHO, which says that health is the height of physical, mental, and social well-being and not only lack of illness or disability [25]. As early as in the 1960s, while referring to the term “disease”, differentiation was introduced on the basis of social sciences into disease, illness, and sickness. The three perspectives of the term were highlighted: medical, individual, and social [26]. Therefore, the phrase “to be healthy” can have different meanings for various groups of people. This can result from different health conditions, as well as, among other things, patients’ gender, age, and current health status. Hierarchy of values of a patient plays a significant role, including the position of health [27]. The assessment of the system of values that seniors attribute with particular dimensions of health is crucial in the face of a systematic increase of their number [28].

The evaluation of health among elderly persons suffering from OA is an innovative study, as such analyses have not been performed before in other countries. For this particular study, we used our self-designed questionnaire and the List of Health Criteria. The survey, administered to a group of patients using the services of rehabilitation outpatient clinics, showed that the respondents defined their state of health as average. The latest European Health Interview Survey confirmed that, because seniors determined their health “as such”, i.e., neither good nor bad. This average assessment was provided by as many as 43% of the respondents. The next group constituted those assessing health as bad and very bad (29%). The last group (28%) included good and very good assessments of health. The EHIS indicated dependence between health assessment and age—it decreases with age. Evidence is provided by surveys among sixty-year-olds and eighty-year-olds. Every third person from the former group claimed to have good and very good health. Only every fifth person was of the opinion that his or her health was bad or very bad. Different tendencies were found in the latter group, since every second respondent assessed his or her health as bad and very bad. Unfortunately, only every eighth individual in the group of eighty-year-olds admitted that his or her health was at least good [29]. Interesting data is provided by a work by the OECD entitled Health at a Glance: Europe 2016 State of Health in the EU Cycle. It says that, in regard to rate of the perceived general health among individuals of 65 years old and more, Poland is found at the end of the list of the membership countries and below the average for the OECD (Poland = 16.6, OECD — 34 = 44.5). The relation between health status and patients’ income, as well as means devoted to healthcare, in each country of the OECD, was highlighted [15]. Therefore, the results of the report of the European Commission entitled the state of health in the EU Poland. The profile of the healthcare system in 2017 reported that fewer Polish patients admitted that their health is good in comparison to most EU countries. Some differences in self-assessment of health and level of income were also indicated: 71% of respondents with income in higher quintile showed good health in comparison with 53% people with lower income. As many as 58% of Poles replied showing good health; though, there were much lower values than the mean for the whole European Union that amounted to 67% [30].

Kurowska and Błasik [31] conducted a study involving elderly persons suffering from chronic diseases. The patients suffering from chronic diseases associated health mainly with not experiencing any ailments (M = 4.0) and feeling happy for most of the time (M = 3.18), i.e., health understood as a property and a state. The lowest number of responses was recorded in relation to health as a process, which is associated with one’s own engagement and health-enhancing behavior patterns. Suffering from a disease is an unpleasant experience for patients, as it evokes numerous emotions and leads to negative consequences. Diseases often result in physical limitations, as well as withdrawal from family life and existing social roles. Hence, it seems justifiable that persons suffering from chronic diseases identify the state of health as not experiencing any ailments. Similarly, our studies conducted on a group of persons suffering from osteoarthritis proved that health was associated largely with a property; the respondents understood it as not experiencing any ailments, having all the body parts functional, or not having to see a doctor. The physical health criteria are related to the instrumental approach to health, understood as means to an end, which is remaining in good health.

Wallston et al. [32] conducted surveys, using the Multidimensional Health Locus of Control on a group of 766 adults. The analysis of variance of MHLC results in relation to three age groups, and the comparison of the types of health locus of control showed that internal control (control of one’s health) decreased with age, while the significance of the influence of others (doctors, medical staff) and chance events increased (*p* < 0.010). In addition, the results of studies conducted on groups of healthy persons, persons suffering from diseases, pregnant women, and menopausal women showed that Polish samples of healthy persons scored higher than American ones in terms of the influence of others and internal locus of control [23,32]. Our studies showed that the majority of persons aged over 60 undergoing treatment for osteoarthritis demonstrated approaches marked by the sense of control over one’s health—health was the outcome of individual behavior patterns, aimed at maintaining health [33]. To a smaller extent, health was seen as the outcome of the actions of other people, in particular those of the medical staff. These actions may include, medical care throughout the entire treatment process, including house calls, health-prevention advice, and doctors’ recommendations related to healthy lifestyle (diet, physical activity, and avoiding psychoactive substances).

As the literature shows, solely Macander et al. [34] conducted a study on the health locus of control among patients with OA being provided with rehabilitation. They were being provided with rehabilitation for hip and knee degenerations, at the municipal outpatient clinic in Łódź for almost two weeks (10 appointments). Among other things, the following tools were used: the acceptance of illness scale (AIS), the 36-Item Short Form Health Survey (SF-36) assessing the quality of life of the sick, and the MHLC. The mean level of quality of life in the patients’ population included in the study was 88.36, which is an average value. The analysis of the results of individual health locus of control scales showed an even distribution among the study participants. The Pearson’s correlation coefficient applied in the study proved that the quality of life was positively correlated with the acceptance of illness and internal locus of pain control in patients. As authors demonstrate, values of scales obtained were similar to those of the previous surveys performed among patients with chronic diseases—American studies showed, respectively, IHLC M = 25.78, PHLC M = 22.54, and CHLC M = 17.648. In the aforementioned study, the Pearson correlation coefficient proved that quality of life diversifies significantly IHLC at the level of *p* < 30. IHLC is favorable and associated with activities directed at improvement of quality of life to date. This results in the promotion of a healthy lifestyle, as well as aspirations for achieving better health and greater autonomy. As Macander et al. show, an individual’s own control over health determines quality of life among patients treated for hip and knee degeneration [33,34]. In our studies, women were more likely to believe that chance events influenced their health, whereas men thought they owed their state of health to their own actions.

Trevisan et al. [35] conducted a cross-section study that was aimed at assessment of the relationship between pain sensation, clinical outcome, and subjective satisfaction, as well as multidimensional health locus of control (MHLC). Mean values of the study for all the respondents for the MHLC dimensions were IHLC 25.3, EHLC 23.5, and CHLC 18.9. Physiotherapy was initiated one day following surgery. Hip functions were assessed by means of the HHS preoperatively and postoperatively. Mean duration of observation was 19.8 ± 8.47 of a month. MHLC was checked in every patient before an operation, and the results were dependent on HHS values. Correlation between patients’ satisfaction and MHLC dimensions showed statistically significant differences on the level *p* < 0.02 only for IHLC; therefore, correlations between pain sensation and MHLC scale indicated average statistical significance (*p* < 0.04), only in relation to IHLC scale in the case of patients with lower pain sensation. Moreover, dependence (*p* < 0.04) between higher values of the postop HHS and IHLC scale. Trevisan et al. suggest further consideration of MHLC as a predictor of satisfaction in patients after total hip endoprosthesis. The authors underlie higher effectiveness of medical care in patients with dominant EHLC.

Wolniak and Skotnicka-Zasadzień [24,36] presented in their publications the results of studies conducted with the use of the modified Serfperf method. They proved that the most deficient areas of outpatient medical care were outdated and unclear websites, nonrenovated clinic buildings, obsolete medical equipment, and insufficient accessibility of the clinic for persons with disabilities. Wolniak and Skotnicka-Zasadzień also analyzed the relationship between the assessment of a given outpatient clinic in relation to other healthcare facilities, as well as between the frequency of using the services and the perception of the 14 variables of the modified Serfperf questionnaire. The analysis of the respondents’ answers demonstrated that a higher assessment of a given outpatient clinic in relation to others determined higher rankings of questionnaire variables. The study showed that the perceived quality of medical services was not only the outcome of an objective assessment of services rendered in a given outpatient clinic, but also of the comparison with the quality of services provided in other entities. We examined whether there were any statistically significant correlations between patients who visited a given outpatient clinic once a week and patients who used its services less than once a year. It was proven that the correlations with a statistic significance of *p* < 0.05 were found in relation to the following variables: opening hours, cleanliness in the outpatient clinic, the appearance of the clinic’s building, the provision of emergency services, and the understanding and patience of the staff toward patients. The analysis of our studies showed a statistically significant, positive correlation between the assessment of the outpatient clinics as compared to other healthcare facilities and the individual dimensions of the Serfperf. The better the assessment of a given outpatient clinic in relation to other healthcare facilities, the higher the score in all the assessment dimensions (quality of service, physical aspects, e-clinic, and location). Patients’ perception of their outpatient clinic from the perspective of other healthcare facilities had a huge influence on the assessment of existing medical services.

Kurowska and Horodecka [37] assessed the health expectations of patients visiting the Non-Pubic Healthcare Centre AGAMED. The study showed that the respondents gave the average score of 7.5 to medical care. The highest scores were given by patients aged over 50 (8.27). The lowest values were recorded among the youngest patients (under 30–7.22). High scores were also found among persons with primary and vocational education (8.8 points). The lowest scores were given by persons with higher education (6.8 points). Residents of rural areas assessed medical care higher by 1.14 point than residents of urban areas. Women proved to have significantly higher expectations than men. The statistical analysis showed significant variations between men and women in terms of the physical-aspects dimension of Serfperf (i.e., the accessibility of the outpatient clinic for persons with disabilities, cleanliness in the outpatient clinic, the appearance of the clinic’s building). Women assessed their respective outpatient clinics higher in the physical aspect than men, which may have stemmed from women’s stronger focus on aesthetics. Also, residents of rural areas assessed outpatient clinic in terms of physical aspects higher than residents of urban areas. This could be related to a limited number of such facilities in rural areas and thus an insufficient number of clinics which might have served as a point of reference, as well as higher expenditures on individual facilities. Residents of urban areas assessed their respective outpatient clinics in terms of their websites higher than residents of rural areas. This may be attributable to a well-developed online infrastructure of city-based rehabilitation outpatient clinics (online booking and the existence and clear layout of the website).

A study of the quality of medical services rendered in outpatient clinics was also conducted by Wyszkowska [38]. The results showed that half of the patients assessed the waiting time as unsatisfactory (50%). Only six respondents (3%) out of the overall number of study participants assessed the waiting time as very good, and 45 respondents (20%) as good. The procedure employed in the present study showed that both the waiting time and the frequency of using outpatient specialist medical services depended on the current waiting list, the dates of therapeutic rehabilitation prescribed by specialists, and the dates of the planned treatment procedures. Detailed information can be found in reports prepared by the WHC Foundation (WHC Barometers), statistical data provided by the National Health Fund, including the appointment date information service, which is a new tool provided by National Health Fund, or data provided by Statistics Poland.

Oleszczyk et al. [39] presented results concerning quality of primary healthcare in Poland assessed by patients. The study was conducted within an international project called the Quality and Costs of Primary Care in Europe (QUALICOPC), in cooperation with 32 European countries, Canada, New Zealand, and Australia. To check quality perceived by the respondents, some areas referring to the process and result were assessed (e.g., continuity, versatility, effectiveness, availability, and equality of care). The questionnaires enabled comparison of respondents in terms of experience in using primary care, including appointments with general practitioners (GP) and care in the following dimensions: availability, continuity, versatility, coordination, quality of service, equality, and effectiveness. As far as expectations and values of different aspects of primary care were concerned, doctor–patient communication and relationship were assessed, along with care in the following dimensions: availability ad continuity, quality of service, and equality. The study on “experience” showed that the highest assessments were provided with continuity (95%), extensiveness (955), and availability of care (94%). Positive predictors of satisfaction in particular dimensions were a very good self-assessment of health with reference to bad health state (*p* < 0.001), GPs’ practice of 5 to 10 years in comparison to shorter practice (*p* < 0.001), and fairness in the provision of services in relation to the place of providing a service (urban or rural) (*p* < 0.001). It was noticed that very good health status influences lower assessment of continuity and effectiveness and better assessment of availability. Thus, patients with chronic diseases assess continuity and coordination higher. Older age was found to diminish risk of negative experiences in all the dimensions, similar to a good doctor–patient relationship [40]. Similarly, Droz et al. [41] obtained results among Norwegian patients, and the highest assessment was given to coordination, continuity, and communication. The last two have a close relationship with presence of chronic diseases. Communication during appointments was of great significance in surveys conducted in Canada and Greece.

Advantages of the analyses conducted include distinguishing individuals with OA among all patients treated at outpatient rehabilitation clinics. Literature shows substantial deficits in the area. Thus, according to studies conducted worldwide, the sample-group patients older than 60 show effectiveness of the analyses in terms of both pathogenesis of the disease and current demographic changes. The results obtained along with conclusions, mainly those regarding development and creation of geriatric rehabilitation outpatient care, demonstrate need for future directions of medical interventions within primary care.

### Study Limitations

The weakness of this study is the lack of references to international sources on the health expectations of patients suffering from OA, as there are no such analyses available. Our study was conducted on the Polish population. In its course, a research tool was used which was not widespread outside Poland. This constitutes a significant limitation in the scope of comparisons in the understanding of health by respondents; however, the authors were limited by the availability of the tool—they did not have an English-language research tool validated to Polish conditions. Undoubtedly, the limitations of this study also included the queues of patients waiting for an appointment with a specialist, the opening hours of the outpatient clinics, and the patients’ functional deficiencies. Considerable lack of appropriately developed medical infrastructure of geriatric profile was noticed that results in a “vicious circle” of expenses, advantages, and needs for medical services. There is massive demand for continuing surveys among patients with OA at the level of outpatient medical care, surveys of patients with OA who are provided with other forms of outpatient care in Poland because of lack of such studies. Questionnaires should take into account patients’ functional restrictions: lengthening the time devoted to filling-in tools, adopting questionnaires in terms of contents and forms of the text, and as marking appropriate answers (considerable difficulties had patients with degenerative changes in their hands). Research on infrastructure of specialist outpatient care where patients with OA are treated and the search for new architectural solutions are requirements that are of great importance in the current medical-care system.

## 5. Conclusions

The considerable majority of the respondents treat health as a value and understand it as lack of ailments, as well as living up to late old age. The respondents were characterized by instrumental comprehension of health (as property) and for most of them, undisturbed physical performance (an ability of independent functioning) was a determinant of optimal health status.

The respondents paid most attention to the appearance of the medical staff as a factor that is evaluated in a subjective way most easily.

The assessment of medical services at outpatient rehabilitation clinics determines the perception of health by the respondents. The more frequently they utilize services in clinics, the higher their conviction of professionalism of the medical staff and the quality of services provided.

Deficits in health status are explained by a negative influence of individuals from the respondents’ environment and an effect of coincidence.

## Figures and Tables

**Table 1:** Assessment of an outpatient clinic in terms of four general dimensions.

Servperf	M	Me	SD
The overall quality of service	3.99	4.00	0.53
Physical aspects	3.93	4.00	0.51
E-clinic	3.89	4.00	0.79
Location	3.65	4.00	0.98

M—mean, Me—median, SD—standard deviation.

**Table 2:** Assessment of an outpatient clinic in terms of particular aspects.

Servperf	M	Me	SD
Neatness (cleanliness) of the medical staff	4.38	4.00	0.55
Cleanliness in the outpatient clinic	4.21	4.00	0.62
Opening hours of the outpatient clinic	4.17	4.00	0.66
Politeness of the medical staff	4.16	4.00	0.71
Willingness of the medical staff to help	4.09	4.00	0.78
Expertise of the medical staff, knowledge of the medical staff	4.07	4.00	0.70
Conscientiousness of the medical staff toward patients	4.04	4.00	0.76
Understanding and patience of the medical staff toward patients	4.02	4.00	0.68
Assessment against other outpatient clinics	4.02	4.00	0.62
Readability of the outpatient clinic’s website	3.89	4.00	0.79
Medical equipment available	3.79	4.00	0.75
Adapting the outpatient clinics to the needs of disabled patients	3.70	4.00	0.87
The proximity of the outpatient clinic to patients’ place of residence	3.65	4.00	0.98
Emergency services provided in the outpatient clinic	3.53	4.00	0.80
The appearance of the clinic’s building	3.42	3.00	0.82

M—mean, Me—median, SD—standard deviation.

**Table 3:** Correlations between general health dimensions and health locus of control.

LHC * MHLC	IHLC		PHLC		CHLC	
	Pho S	*p*	Rho S	*p*	Rho S	*p*
State	−0.146	0.008 **	−0.046	0.407	0.023	0.683
Purpose	0.194	0.000 ***	0.178	0.001 ***	0.033	0.556
Process	−0.045	0.419	0.168	0.002 **	−0.015	0.787
Property	−0.179	0.001 **	−0.202	0.000 ***	0.028	0.618
Outcome	0.272	0.000 ***	0.027	0.624	0.001	0.992

LHC—List of Health Criteria, MHLC—Multidimensional Health Locus of Control Scale, IHLC—Internal Health Locus of Control, PHLC—External Health Locus of Control, CHLC—Chance Health Locus of Control, Rho S—correlations of Spearman’s rho, *p*—statistical significance, * *p* < 0.05, ** *p* < 0.01, *** *p* < 0.001.

**Table 4:** Correlations between particular health criteria and health locus of control.

LHC * MHLC	IHLC		PHLC		CHLC	
	Rho S	*p*	Rho S	*p*	Rho S	*p*
To live up to old age	0.212	0.000 ***	0.231	0.000 ***	0.094	0.088
To feel happy most of the time	−0.012	0.828	0.004	0.941	0.077	0.162
To be able to get on well with people	−0.035	0.531	0.152	0.006 **	0.023	0.673
To be able to solve one’s own problems	−0.069	0.210	0.089	0.107	0.139	0.012 *
To follow a healthy diet	0.153	0.005 **	0.071	0.197	−0.064	0.250
To make sure to get enough rest and sleep	0.261	0.000 ***	−0.045	0.415	−0.045	0.421
To drink little or no alcohol	0.121	0.029 *	0.101	0.067	0.151	0.006 **
Not to smoke cigarettes	0.192	0.000 ***	0.002	0.975	−0.011	0.845
To have an appropriate body weight	0.020	0.723	−0.067	0.229	−0.057	0.305
To take medications only exceptionally	−0.038	0.492	−0.074	0.182	0.140	0.011 *
To be in a good mood	0.011	0.836	0.033	0.546	0.170	0.002 **
Not to experience any ailments	−0.070	0.206	−0.109	0.048 *	−0.080	0.149
To be able to work without tension and stress	0.088	0.111	0.147	0.008 **	−0.133	0.016 *
Not to get sick, except for occasional flu	−0.198	0.000 ***	−0.141	0.011 *	0.009	0.871
To have healthy eyes, hair and complexion	−0.045	0.413	0.101	0.068	0.107	0.054
To be able to adapt to changes in life	−0.119	0.032 *	0.019	0.729	−0.112	0.043 *
To be able to enjoy life	−0.086	0.118	0.062	0.260	−0.029	0.606
To be responsible	−0.025	0.650	−0.155	0.005 **	−0.068	0.222
To be able to control one’s own emotions	−0.052	0.344	−0.006	0.917	−0.102	0.066
To have all parts of the body functional	−0.225	0.000 ***	−0.131	0.018 *	−0.145	0.008 **
To accept oneself, to know one’s own abilities and deficiencies	−0.014	0.799	0.069	0.212	−0.042	0.445
To have a job, varied interests	−0.038	0.493	0.028	0.618	−0.047	0.394
To feel good	−0.188	0.001 **	−0.082	0.140	−0.065	0.239
Almost never to have to see a doctor	0.060	0.276	−0.220	0.000 ***	0.083	0.134

LHC—List of Health Criteria, MHLC—Multidimensional Health Locus of Control Scale, IHLC—Internal Health Locus of Control, PHLC—External Locus of Control, CHLC—Chance Health Locus of Control, Rho S—correlations of Spearman rho, *p*—statistical significance, * *p* < 0.05, ** *p* < 0.01, *** *p* < 0.001.

**Table 5:** Correlations between particular health criteria and satisfaction with services provided by an outpatient clinic.

LHC * SP	Total Quality of Service		Physical Aspects		E-Outpatient Clinic		Localization	
	Rho S	*p*	Rho S	*p*	Rho S	*p*	Rho S	*p*
To live up to old age	−0.032	0.567	0.105	0.057	0.093	0.268	0.092	0.095
To feel happy most of the time	0.001	0.989	−0.076	0.168	−0.081	0.334	0.009	0.869
To be able to get on well with people	−0.017	0.765	0.015	0.791	0.228	0.006 **	−0.016	0.767
To be able to solve one’s own problems	−0.162	0.003 **	−0.193	0.000 ***	−0.055	0.514	0.015	0.790
To follow a healthy diet	−0.025	0.658	0.053	0.335	0.090	0.286	0.057	0.301
To make sure to get enough rest and sleep	0.031	0.576	0.088	0.113	0.103	0.220	0.158	0.004 **
To drink little or no alcohol	−0.257	0.000 ***	0.022	0.697	−0.212	0.011 *	0.015	0.786
Not to smoke cigarettes	−0.124	0.024 *	0.105	0.058	−0.266	0.001 **	−0.036	0.521
To have an appropriate body weight	−0.006	0.913	0.041	0.459	−0.095	0.260	0.083	0.136
To take drugs only exceptionally	0.052	0.346	−0.031	0.576	0.095	0.261	−0.051	0.358
To be in a good mood	−0.073	0.188	0.001	0.985	−0.079	0.348	−0.065	0.239
Not to experience any ailments	0.010	0.850	−0.229	0.000 ***	−0.172	0.040 *	−0.071	0.199
To be able to work without tension and stress	0.017	0.759	0.040	0.470	0.107	0.204	0.231	0.000 ***
To to get sick only, except for occasional flu	0.014	0.802	0.025	0.648	−0.142	0.090	−0.116	0.036 *
To have healthy eyes, hair and complexion	0.089	0.109	0.152	0.006 **	0.075	0.375	0.001	0.980
To be able to adapt to changes in life	−0.049	0.380	0.068	0.216	−0.220	0.008 **	0.062	0.265
To be able to enjoy life	0.178	0.001**	0.211	0.000 ***	0.332	0.000 ***	−0.005	0.935
To be responsible	0.028	0.619	0.018	0.744	−0.042	0.620	0.087	0.114
To be able to control one’s own emotions and drives	0.015	0.781	−0.004	0.945	0.016	0.853	0.053	0.339
To have all parts of the body functional	0.155	0.005 **	0.021	0.708	0.053	0.530	−0.076	0.169
To accept oneself, to know one’s own abilities and deficiencies	0.224	0.000 ***	−0.185	0.001 **	0.199	0.017 *	−0.146	0.008 **
To have a job, varied interests	0.027	0.628	−0.006	0.917	0.019	0.820	0.010	0.853
To feel good	−0.117	0.034 *	−0.063	0.254	−0.018	0.828	−0.113	0.041 *
Almost never to have to go and see a doctor	0.031	0.578	−0.075	0.173	−0.112	0.181	−0.042	0.449

LHC—List of Health Criteria, SP—Service Performance, MHLC—Multidimensional Health Locus of Control Scale, IHLC—Internal Health Locus of Control, PHLC—External Health Locus of Control, CHLC—Chance Health Locus of Control, Rho S—correlations of Spearman’s rho, *p*—statistical significance, * *p* < 0.05, ** *p* < 0.01, *** *p* < 0.001.
